# Use of antenatal services and delivery care in Entebbe, Uganda: a community survey

**DOI:** 10.1186/1471-2393-7-23

**Published:** 2007-10-11

**Authors:** Carolyn J Tann, Moses Kizza, Linda Morison, David Mabey, Moses Muwanga, Heiner Grosskurth, Alison M Elliott

**Affiliations:** 1 London School of Hygiene & Tropical Medicine, Keppel Street, London, WC1E 7HT, UK; 2 Uganda Virus Research Institute (UVRI), PO Box 49, Entebbe, Uganda; 3 Medical Research Council (MRC)/UVRI Uganda Research Unit on AIDS, PO Box 49, Entebbe, Uganda; 4 Entebbe Hospital, Entebbe, Uganda

## Abstract

**Background:**

Disparities in perinatal health care occur worldwide. If the UN Millennium Development Goals in maternal and child health are to be met, this needs to be addressed. This study was conducted to facilitate our understanding of the changing use of maternity care services in a semi-urban community in Entebbe Uganda and to examine the range of antenatal and delivery services received in health care facilities and at home.

**Methods:**

We conducted a retrospective community survey among women using structured questionnaires to describe the use of antenatal services and delivery care.

**Results:**

In total 413 women reported on their most recent pregnancy. Antenatal care attendance was high with 96% attending once, and 69% the recommended four times. Blood pressure monitoring (95%) and tetanus vaccination (91%) were the services most frequently reported and HIV testing (47%), haematinics (58%) and presumptive treatment for malaria (66%) least frequently. Hospital clinics significantly outperformed public clinics in the quality of antenatal service. A significant improvement in the reported quality of antenatal services received was observed by year (p < 0.001). Improvement in the range and consistency of services at Entebbe Hospital over time was associated with an increase in the numbers who sought care there (p = 0.038). Although 63% delivered their newborn at a local hospital, 11% still delivered at home with no skilled assistance and just under half of these women reported financial/transportation difficulties as the primary reason. Less educated, poorer mothers were more likely to have unskilled/no assistance. Simple newborn care practices were commonly neglected. Only 35% of newborns were breastfed within the first hour and delayed wrapping of newborn infants occurred after 27% of deliveries.

**Conclusion:**

Although antenatal services were well utilised, the quality of services varied. Women were able and willing to travel to a facility providing a good service. Access to essential skilled birth attendants remains difficult especially for less educated, poorer women, commonly mediated by financial and transport difficulties and several simple post delivery practices were commonly neglected. These factors need to be addressed to ensure that high quality care reaches the most vulnerable women and infants.

## Background

Great disparities in perinatal health occur worldwide [1]. Between 7 and 8 million perinatal deaths occur globally each year with the majority occurring in developing countries where over 90% of all infants are born [2]. Over recent decades, infant mortality rates have fallen, but stagnating neonatal mortality rates mean that deaths in the newborn period are becoming responsible for an ever-increasing proportion of infant and child mortality [3]. If the Millennium Development Goals in maternal and child health aimed at reducing child mortality, are to be met this will need to be addressed [4]. In Uganda, global trends in infant and neonatal mortality have been mirrored: nationally, neonatal mortality rates (NMR) remain high (33 per 1000 live births) and deaths in the newborn period are now responsible for 40% of all deaths occurring under one year of age [5]. National perinatal mortality rates (PNMR) are estimated to be between 43 and 70 per 1000 births [5,1] and audit data from the local government hospital in Entebbe reflects this with a PNMR of 56 per 1000 (unpublished data) although selection bias at the hospital towards high-risk pregnancies and deliveries may mean that mortality in the community overall is lower. Often however, the true burden of neonatal morbidity and mortality is concealed due to delays and difficulties in presentation for care and the relative speed in which newborns can succumb to infection or perinatal hypoxia. In addition, newborn deaths occurring in the community may often go unreported.

Attendance for antenatal care represents a unique opportunity to improve the health of women and their infants. It is imperative that we optimise this opportunity by offering a full range of health promoting services that may include voluntary counselling and testing for HIV (VCT), screening and treatment for syphilis, prevention and presumptive treatment of malaria in pregnancy (IPTp) and health education. At delivery, the importance of skilled attendance has long been recognised. However, distance to health facilities, inadequate transportation and the need for immediate and specialised services have hampered women's ability to access these services [2]. Attention to clean and hygienic delivery practices [6] and the provision of essential care for the newborn, such as thermal protection and early and exclusive breast-feeding [7], are important for the health of all infants whether born at home or in a health care facility. Community and primary care level interventions targeting the reduction of maternal and neonatal mortality have been found to be highly cost effective, although in many communities these services are still lacking [8]. In Uganda, the national Maternal Mortality Ratio is estimated to be 505 per 100,000 live births [5]. Levels of antenatal care attendance are high (94%) but this is not reflected in care at delivery. Around a quarter of women are assisted during labour by a relative or friend and one in seven mothers receive no assistance at all [5].

The aims of this study were to describe the changing use of antenatal services and delivery care amongst women living in a semi-urban community in and around Entebbe in central Uganda; to facilitate our understanding of women's access to antenatal and maternity services; to examine the range of services that they received, and to describe common perinatal care practices that occur both at home and in health care facilities. The study was conducted in preparation for research on, and implementation of, new measures to improve delivery outcomes and neonatal survival in this region.

## Methods

### Setting

**Figure 1:**
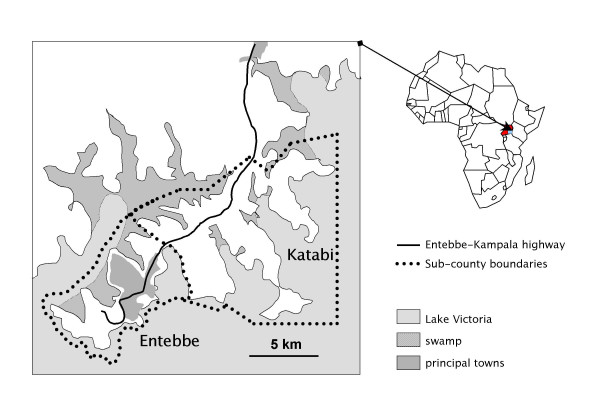
Study area.

The survey was carried out amongst the community of Entebbe Municipality and Katabi Subcounty in central Uganda. The study area comprises 9 parishes divided into 47 wards (each with an elected local executive council) and 5 governmental army barracks. The geographical terrain of the study area is diverse. Entebbe Municipality forms a peninsula projecting into Lake Victoria and is located around 40 km Southwest of the Ugandan capital, Kampala. It is transected by Entebbe International Airport. Katabi Subcounty borders Entebbe and extends either side of the Entebbe-Kampala road. The Katabi community combines the semi-urban population within close proximity to the road and the relatively isolated rural fishing communities residing on small peripheral peninsulas extending out into the waters and marshland of Lake Victoria (Figure 1).

Entebbe General Hospital, the major provider of governmental health services in the area, is located centrally within Entebbe town. In 2002, in accordance with Ministry of Health policy, a programme for prevention of mother-to-child HIV transmission (PMTCT) was established by Entebbe Hospital in collaboration with a research study examining the effect of helminth infections on the response to immunisations in childhood (clinical trial registration: ISRCTN32849447). In addition to training, the ongoing research study employed counsellors to facilitate entry into the PMTCT programme and a laboratory technician to perform rapid antibody testing for HIV and syphilis. Women attending the clinic are routinely offered iron and folate supplementation and intermittent presumptive treatment for malaria in pregnancy (IPTp). Surveillance data from the hospital estimates the prevalence of HIV amongst women attending for antenatal care to be around 12%, while that of active syphilis was less than 2% [9]. A number of other health facilities in the area offer maternity services. These include small government health centres (public clinics); private delivery homes owned and run by retired midwives, and private hospitals.

### Study Design

We conducted a cross-sectional retrospective community survey to identify experiences of maternity care and practice for each pregnancy within the past five years. Consent to conduct the survey was obtained from local community leaders and participating women gave verbal consent to be involved. The study was reviewed and approved by the ethics committee at the London School of Hygiene and Tropical Medicine, the Ugandan Virus Research Institute (UVRI) Science and Ethics Committee and the Ugandan National Council for Science and Technology.

### Sampling Strategy

Wards were chosen as the sampling unit and census statistics (Ugandan Bureau of Statistics) were used to estimate the relative population size of each ward. A sample of 40 wards was randomly selected, by probability proportional to size, and we aimed to select 16 households within each ward, expecting that on average two thirds of these households would have at least one woman who had given birth over the last 5 years. Maps were available for the study area with the location of major roads and landmarks but not the location of individual households. Each ward was divided into segments of equal geographical size (approximately 70 m^2^) and four segments were randomly selected from each ward (ranging between 99 and 641 segments per ward). The centre point of the segment was used to define the starting point for the sampling of 4 households. The first household was the household closest to the start point. Each subsequent household was selected as the closest to the one before it.

The head of selected households was approached by a pair of interviewers and asked about the presence of any women in the household who had had a pregnancy within the past five years. No exclusion criteria were applied and all women who reported a pregnancy in the past five years were invited to respond. If any such person was identified, verbal consent was obtained before administration of the survey questionnaire. Where such a woman was resident but not present at that time, interviewers returned to the property on at least one occasion at a later date. Where a woman was unavailable due to working or travel, interviewers made every effort to return at a time when she might be expected to be at home (such as the evening or weekend). We were unable to use antenatal cards and delivery records to crosscheck information because women do not routinely keep these after delivery and they are often retained by health facilities.

### Data collection

Structured questionnaires were designed to identify the antenatal, delivery and postpartum experiences of women. Questionnaires were administered in the local language by pairs of interviewers, with all pairs containing at least one female interviewer. Answers were predefined with tick boxes or were described under 'other'. The questionnaire and sampling strategy within households were pre-tested in a ward not selected for inclusion into the survey. Women were asked to answer questions with regard to each pregnancy experienced within the past five years, starting with the most recent. Information sought regarding antenatal care during each pregnancy included the frequency of antenatal visits, the primary place of attendance and the type of services that they received there. In addition, women were asked about their delivery experience for each pregnancy including the place of delivery, what assistance they received during labour, hygiene practices at birth and care of the infant soon after delivery.

### Statistical Methods

Data were entered and checked using Excel (Microsoft) and analysed using Stata version 8 (StataCorp, Texas). Analysis relating to pregnancies was restricted to the most recent pregnancy to minimise recall bias and to avoid using a complicated hierarchical analysis to take into account correlation within woman. A negligible number of households contained more than one eligible woman, so the only level of clustering taken into account in the analysis was that within wards. This was done using the complex survey commands within stata. Initial comparisons were made using simple tables (svytab). To assess associations between demographic or socio-economic variables and antenatal or delivery/perinatal care practices, binary outcome variables were developed and associations examined using logistic regression models (svylogit) to adjust for possible confounding variables. To obtain an overall assessment of the quality of antenatal services and postnatal practices at delivery, scores were developed for this study. One point was given for each service received of the seven listed in table 1 for antenatal care, and one point for each of the postnatal practices considered to be beneficial among the six listed for postnatal practices. Delivery practices were not included in the scores as it was felt that these might be inaccurately recalled; further, over 90% of women reported that appropriate procedures had been used, so that these parameters would not discrimate well between comparison groups in an overall assessment. For each score a binary variable was created with values below the median defined as low score, including and above the median as high score.

**Table 1:** Antenatal services and delivery/perinatal practices during the most recent pregnancy among 413 Ugandan women

	Percent of mothers
Number evaluated for antenatal services received	n = 355

Setting in which antenatal services were received	
Entebbe Hospital	60%
Other government hospitals	15%
Private hospital	15%
Public clinic	10%

Gestation at first ANC visit	
First trimester	16%
Second trimester	65%
Third trimester	19%

Number of antenatal visits	
1 visit	4%
2–3 visits	27%
4–6 visits	39%
6–10 visits	25%
>10 visits	5%

Services received	
Blood pressure check	94%
Intermittent presumptive treatment for malaria (IPTp)	66%
Counselling and offer of HIV testing	47%
Syphilis test	68%
Haematinics	58%
Tetanus toxoid	91%
Antenatal education	69%

Number evaluated for delivery/perinatal practices	n = 328

Setting in which delivery was conducted	
Entebbe Hospital	38%
Other government hospital	12%
Private hospital	13%
Public clinic	20%
Traditional birth attendant	5%
At home; no trained assistance	11%

Delivery practices	
Assistant used gloves/washed hands with soap	95%
Delivery onto a clean surface	93%
Cord tied with hospital tie or new/clean thread/cloth	98%
Cord cut with new blade or hospital scissors	99%

Postnatal practices	
Cord dressed with nothing/spirit/antiseptic	77%
Baby wrapped before placenta delivered	73%
Baby first bathed after at least 24 hours	30%
Baby offered to breast-feed in < 1 hour	35%
Baby given colostrum	98%
Baby given breast-milk as its first feed	60%

## Results

The survey was conducted over a six-week period commencing on the 15^th ^March 2004. The study design predicted that 640 households would be visited, but some of the selected segments contained less than 4 inhabited homes; thus data were collected for 610 households only. Within these households 410 had one woman who had been pregnant in the last 5 years; 15 had two such women. Of these 440 women, 413 (94%) were available and gave information. The median time from interview to delivery date was 2.25 years. Complete data sets in each area were difficult to obtain as at times women were unable to recall, or gave no responses with regard to occasional variables, however the relevant missing data are indicated in the following results.

Among the 413 women who responded to questions about their most recent pregnancy the mean age was 26 years; the majority (78%) were married or living as married and 71% were housewives with no formal occupation; 12% had received no formal education, 49% some primary education and 39% some secondary or tertiary education. Most were poor, with low scores for socioeconomic parameters such as items owned in the family (64% none of the items listed or a bed only; 30% a mobile phone, motorbike or car). Of these 413 women, 28 were unable to recall, or gave no response regarding frequency of antenatal clinic attendance, providing 385 respondents (88% of all women in the household sample). Of these, 369 (96%) had attended antenatal clinic at least once and 355 (92%) at least twice. Of those who attended for antenatal care, 14 were unable to recall, or gave no response with regard to antenatal services received, meaning that complete reports were available for 355 women. Complete reports on delivery and perinatal practices were available for 339 live births. The median antenatal quality score was five (range 0–7) out of seven possible points. The median postnatal quality score was 4 (range 1–6) out of a possible maximum score of six. These binary variables were used to relate services and practices reported to the care setting and to demographic and socioeconomic variables. A considerable number of mothers gave incomplete responses regarding socioeconomic variables and two had missing data regarding their ward, only those with complete information were used in the multivariate the analyses (294 were included for analysis of antenatal care and 270 for analysis of delivery and perinatal practices; 83% and 82% of all mothers respectively).

Table 1 shows the type of facility attended and the services received. Antenatal services were variable with the provision of HIV voluntary counselling and testing, haematinics and IPTp being least frequently reported. Coverage of blood pressure monitoring and tetanus toxoid vaccination was good throughout. For antenatal care, the strongest associations were observed with care setting and year of pregnancy: low scores were observed among mothers attending public clinics rather than hospitals, and a highly significant improvement in services was observed by year (Table 2). Neither effect was explained by adjusting for potentially confounding socioeconomic factors. There was a tendency for younger mothers and better-educated mothers to report better services. These effects were reduced after adjusting for care setting, in part possibly because better-educated mothers were aware that quality of services differed in different settings. All antenatal services listed, except IPTp and haematinics, were significantly less likely to have been given at clinics than at hospitals.

**Table 2:** Factors associated with reported antenatal services during the most recent pregnancy

	Proportion (%) with high score	Crude OR for high score	P value	Adjusted OR^a ^for high score (95% confidence interval)	P value
**Setting**					
Entebbe Hospital	127/184 (69%)	1	0.027^b^	1	0.026^b^
Other government hospitals	22/41 (54%)	0.52		0.63 (0.27, 1.47)	
Private hospital	31/45 (69%)	0.99		0.86 (0.35, 2.13)	
Public clinic	6/24 (25%)	0.15		0.11 (0.03, 0.44)	
Traditional Birth Attendant	none				
**Year of pregnancy**					
1999	23/47 (47%)	1	<0.001^c^	1	<0.001^c^
2000	24/42 (57%)	1.52		1.37 (0.53, 3.56)	
2001	22/48 (46%)	0.96		1.33 (0.50, 3.57)	
2002	40/60 (67%)	2.27		2.16 (0.82, 5.70)	
2003	61/75 (81%)	4.95		5.32 (2.52, 11.23)	
2004 (to March)	17/22 (77%)	3.86		4.78 (1.03, 22.24)	
**Age**					
13–25 years	94/135 (70%)	1	0.041	1	0.15
25 years or more	92/159 (58%)	0.60		0.70 (0.43, 1.14)	
**Gravidity**					
Primigravida	64/94 (68%)	1	0.29	1	0.88
Multigravida	122/200 (61%)	0.73		0.95 (0.51, 1.77)	
**Education**					
None	15/28 (54%)	1	0.015^c^	1	0.044^c^
Primary	87/146 (60%)	1.28		1.14 (0.39, 3.34)	
Secondary	67/98 (68%)	1.87		1.49 (0.53, 4.22)	
Tertiary	17/22 (77%)	2.95		3.11 (0.99, 9.80)	
**Possessions**^d^					
None of specified items/bed	122/196 (62%)	1	0.65	1	0.84
Phone/motorbike/car	64/98 (65%)	1.14		1.06 (0.62, 1.80)	
**Electricity at home**					
No	91/157 (58%)	1	0.13	1	0.32
Yes	95/137 (69%)	1.64		1.43 (0.70, 2.93)	
**Crowding at home**					
3 or more	95/146 (65%)	1	0.44	1	0.34
Less than 3 people/room	91/148 (61%)	0.86		0.81 (0.52, 1.26)	

^a ^Adjusted for anatenatal care setting, year of pregnancy, age-group, mother's education, and electricity in the home. ^b^Test for heterogeneity. ^c^Test for trend. ^d^Specified possessions were bed, mobile phone, motorcycle and car. Crude and adjusted odds ratios (OR) and P values were computed using complex survey commands within Stata (svylogit).

**Figure 2:**
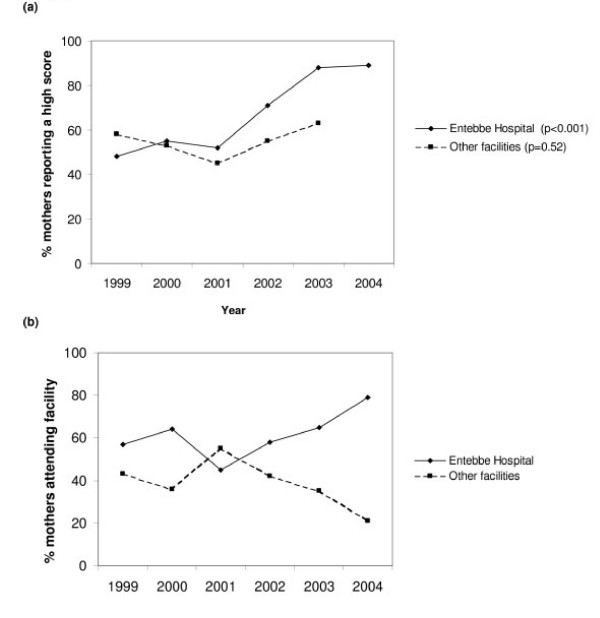
Trends by year in proportion of mothers. (a) reporting a high score for antenatal services at Entebbe Hospital compared with other facilities. (b) attending Entebbe Hospital compared with other facilities. P values for a test for trend were obtained using complex survey commands (svylogit).

**Figure 3:**
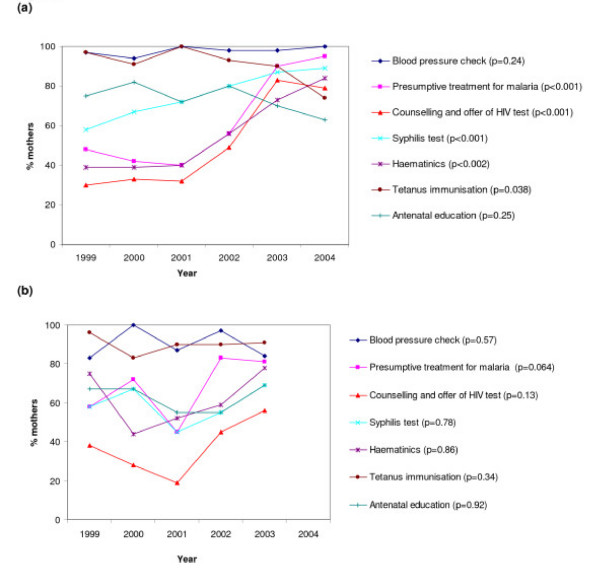
Trends by year in proportion of mothers reporting receipt of specific antenatal care services. (a) at Entebbe Hospital. (b) at other facilities. P values for a test for trend were obtained using complex survey commands (svylogit).

The improvements observed in antenatal care by year were largely explained by improvements over time in care at Entebbe Hospital, the main hospital in the area (Figure 2(a)) and by an increase over time in the proportion of mothers who sought care at Entebbe Hospital (p = 0.038; Figure 2(b)). At Entebbe Hospital, improvements in provision of IPTp, haematinics, counselling and HIV testing and syphilis testing were particularly marked (Figure 3(a)). The provision of tetanus immunisation slightly declined, but not among primigravidae where rates remained at 90% or above (data not shown); tetanus immunisation is not required among multigravidae who have received five doses during previous pregnancies. At other facilities there was no statistically significant trend in overall score with year (Figure 2(a)). There were moderate increases in the provision of IPTp and of counselling and HIV testing but neither was statistically significant (Figure 3(b)). There was no significant change over time in the proportion of women booking by trimester.

For both antenatal and delivery care there were associations between personal and socioeconomic characteristics and care setting. For antenatal care, multigravidae were more likely to attend Entebbe Hospital, compared to other facilities (OR 1.85, 95%Confidence Interval (CI) 1.13–3.00) and better-educated mothers were more likely to go to a private hospital (OR 3.32, 95%CI 1.84–6.00). At delivery, primigravidae were more likely to attend another government hospital (outside the Entebbe area) (OR 2.24, 95%CI 1.12–4.46) and less educated, poorer mothers were more likely to have a TBA or no trained assistant (for less education: OR 3.07, 95%CI 1.49–6.31; for measures of household wealth: lack of electricity OR 3.60, 95%CI 2.04–6.37; ownership of fewer items OR 3.47, 95%CI 1.51–7.97; crowded household OR 2.71, 95%CI 1.42–5.16).

A high proportion of women delivered their infant at a hospital or public clinic but one in 10 women still delivered their infant at home with no trained assistance (table 1). Of these 44 women who delivered at home: 34% (15) stated that this was due to financial limitations, 23% (10) due to transport limitations, and 27% (12) due to 'other reasons' which were most commonly stated as to be due to the delivery occurring too quickly or too late at night to attend the facility of choice. Of the women who reported that financial and transport limitations were the most important factor in where they delivered, 40% (17/42) and 37% (10/27) respectively delivered at home with no trained assistance.

Hygiene practices and cord care at the time of delivery were generally reported as very good but, in the immediate aftermath, delay in wrapping was common and babies were frequently bathed too soon. Untrained assistants were less likely to be reported to have washed their hands with soap and water or used gloves and often delayed the first feeding beyond one hour. Both untrained assistants and traditional birth attendants were less likely to be reported to have used a clean surface for the delivery or to have tied the cord with a clean or new thread; together with clinics, they were reported to have bathed the baby early. Traditional birth attendants were more likely to have applied material other than spirit/antiseptic to the cord stump. Offering the breast was often delayed beyond one hour, and only 78% of infants had been put to the breast within 4 hours of delivery. Forty percent of babies were given something other than breast milk for their first feed, including water, glucose, salt solution or tea (28%); ghee (3%); mushroom soup (2%) or herbs (1%).

For postnatal practices, the strongest association was again with care setting: lower scores were reported by mothers who delivered at public clinics rather than hospitals, and, in addition, by those who delivered with a traditional birth attendant or without trained assistance (Table 3). There was no association between reported delivery practices and year of delivery, but there was a stronger suggestion of an association between good practices and higher socioeconomic status than observed for antenatal care, and multigravidae reported better practices than primigravidae.

**Table 3:** Factors associated with reported postnatal practices following the most recent pregnancy

	Proportion (%) with high score	Crude OR for high score	P value	Adjusted OR^a ^for high score (95% onfidence interval)	P value
Setting					
Entebbe Hospital	78/115 (68%)	1	0.011^b^	1	0.020^b^
Other government hospitals	12/29 (41%)	0.33		0.33 (0.13, 0.83)	
Private hospital	28/39 (72%)	1.21		1.02 (0.41, 2.54)	
Public clinic	24/51 (47%)	0.42		0.34 (0.18, 0.63)	
Traditional Birth Attendant	4/14 (29%)	0.19		0.26 (0.08, 0.82)	
No trained assistance	16/35 (46%)	0.40		0.48 (0.19, 1.19)	
Year of pregnancy					
1999	27/48 (56%)	1	0.83^c^	1	0.63^c^
2000	25/43 (58%)	1.08		1.04 (0.36, 3.06)	
2001	28/47 (60%)	1.15		1.29 (0.56, 3.01)	
2002	28/51 (55%)	0.95		1.08 (0.35, 3.33)	
2003	44/73 (60%)	1.18		1.35 (0.67, 2.72)	
2004 (to March)	10/21 (48%)	0.71		0.94 (0.38, 2.36)	
Age					
13–25 years	66/136 (49%)	1	0.011	1	0.38
25 years or more	96/147 (65%)	1.99		1.30 (0.71, 2.37)	
Gravidity					
Primigravida	40/90 (44%)	1	0.029	1	0.047
Multigravida	122/193 (63%)	2.15		2.18 (1.01, 4.70)	
Education					
None	13/28 (46%)	1	0.12^c^	1	0.70^c^
Primary	80/141 (57%)	1.51		1.25 (0.52, 2.96)	
Secondary	55/96 (57%)	1.55		1.02 (0.45, 2.31)	
Tertiary	14/18 (78%)	4.04		2.21 (0.71, 6.89)	
Possessions^c^					
None of specified items/bed	100/196 (51%)	1	0.004	1	0.054
Phone/motorbike/car	62/87 (71%)	2.38		1.90 (0.99, 3.63)	
Electricity at home					
No	84/157 (54%)	1	0.18	1	0.80
Yes	78/126 (78%)	1.41		1.07 (0.62, 1.84)	
Crowding at home					
3 or more	68/142 (48%)	1	<0.001	1	0.009
Less than 3 people/room	94/141 (67%)	2.18		1.96 (1.19, 3.20)	

^a ^Adjusted for delivery care setting, age-group, gravidity, mother's education, possessions, electricity in the home and crowding. ^b^Test for heterogeneity. ^c^Test for trend. ^d^Specified possessions were bed, mobile phone, motorcycle and car. Crude and adjusted odds ratios (OR) and P values were computed using complex survey commands within Stata (svylogit).

## Discussion

This study aimed to examine the use of antenatal and delivery care amongst women in a semi-urban community in Uganda. We found that antenatal services were well utilised, but that the availability of individual services varied with mothers able and willing to travel a moderate distance to a facility providing a good service. Access to good delivery care appeared more problematic especially for poorer, less educated women. Women who delivered their infants at home with no trained assistance commonly reported financial or transportation difficulties as key factors. Several simple but important delivery and post delivery practices were commonly neglected. Poorer, less educated mothers were less likely to access good quality antenatal and delivery/postnatal care mainly through their choice of care setting.

We know of few studies that have used patients' reports to assess quality of care. One of the strengths of patients' reports is their longitudinal nature and this may to some extent overcome the limitations intrinsic to a cross-sectional survey and the bias that is inherent to assessing service provision and use during announced visits by observers, to individual care centres. On the other hand, it is possible that responses with regard to pregnancies and deliveries occurring up to five years previously were subject to significant recall bias. In addition, recall may differ according to the type of event to which it refers with life events, such as a death, being less subject to bias than more incidental events. For instance, women would perhaps be more likely to accurately recall the place and type of assistance that they received at delivery than whether their blood pressure was monitored during pregnancy or whether their birth attendant wore gloves or washed their hands. Our results presenting the circumstantial events around pregnancy and delivery care must be interpreted with this in mind. However, the consistency seen amongst women's responses with regard to the availability of individual antenatal services, and the consistency of these responses with the known timing of introduction of services at Entebbe Hospital in particular, suggests that women in reality had good recall of events. It is also possible that women may have shown a tendency to report what they assumed to be desired practices although interviewers were trained to impress that there were no 'right' or 'wrong' answers and that all responses were valid, confidential and anonymous.

It was not within the limitations of this survey to directly assess maternal and infant mortality since this would require a much larger sample size. In addition, the assessment of perinatal/neonatal mortality from personal reports of recalled events may be complicated by difficulties in differentiating between a miscarriage & stillbirth (i.e. early & late fetal death) and between a late fetal death and an early neonatal death.

In our study, levels of attendance at antenatal care are high and this reflects national estimates showing high ANC attendance with 94% of women attending at least once [5]. As commonly seen throughout Africa, the majority of women delay presentation for care until the second trimester with only one in 7 attending in the first. Current government policy in Entebbe recommends that women attend for antenatal care four times during their pregnancy but it is vital that these visits include a comprehensive range of services. Our study findings highlight that whilst more than two thirds of women achieved four visits, the quality of the care received varied significantly, with hospitals consistently outperforming clinics. Of concern was the relatively low coverage of IPTp and VCT procedures that must be considered essential in a community where more than one in 10 women are HIV positive [9] and malaria is endemic. It has been shown that high levels of antenatal care can have a positive effect on the use of safe delivery practices [10] and may promote interaction with health services in general to enable families to seek to access care more efficiently [11], suggesting that the importance of good quality care during pregnancy extends beyond the antenatal period. Encouragingly, at Entebbe Hospital, a significant trend can be seen in the improvement of antenatal services over time. A major factor in these improvements has likely been the development of the governmental PMTCT programme and the initiation of a research study in 2002 that enrolled its participants through the hospital antenatal clinic. These activities together ensured consistent provision of simple, essential laboratory services, counselling and basic medications. Perhaps in response to this, significantly more women are now attending the hospital for their pregnancy care.

Providing access to care during the relatively short period of labour and delivery is logistically much more difficult than making services available during the antenatal period [12]. Other studies from East Africa have shown low levels of skilled birth attendance despite high rates of antenatal care coverage [13,14]. The importance of the skilled birth attendant, along with a supporting continuum of secondary and tertiary care, in case birth complications occur, has been recognised [15,16]. Nationally, just over one in 3 women are thought to deliver at a health facility, although this is significantly higher in urban that rural areas (79% vs 32% respectively) [5]. Home delivery with no trained assistance is also considerably higher nationally (43%) than in our population, again with the poorest of women most at risk (54% amongst the poorest women compared to 15% amongst the wealthiest) [5]. Previous studies have identified an urgent need to improve the availability of skilled attendance at delivery in East Africa [13,14,17], and in this semi-urban community the levels of hospital attendance for delivery that we observed were promising (63%). Despite the availability of services however, one in 10 women still went on to deliver at home without any trained assistance, and this effect was greatest for the most vulnerable, less educated, poorer women. Financial and transport limitations appear to be some of the most important factors in women's inability to access skilled care in Entebbe and these factors have been highlighted elsewhere in east Africa [18-20]. This important barrier to care will need to be addressed if we are to improve delivery service to the most vulnerable of women.

However, many of the deficits in perinatal care identified in our study were related to simple procedures that could readily be adopted if mother and attendant are aware and convinced of their importance. Thus, WHO advice on essential care of the newborn encompasses clean delivery and cord care for the prevention of infections, thermal protection for the prevention of hypothermia and early and exclusive breast-feeding [7]. Of these, hygiene practices during labour were reported to be of a high standard in all facilities: hospitals, clinics and with TBA's but other standards of essential postnatal care showed considerably more variation. Recommendations for thermal protection include early drying and wrapping of newborns and delayed bathing. In our survey more than a quarter of babies were not dried and wrapped until after the placenta was delivered, and two thirds of babies were bathed within 24 hours of birth, placing these newborns at a significant risk of hypothermia. In our survey nearly a quarter of women reported application of a potentially non-sterile substance to the cord that may increase the risk of neonatal sepsis and tetanus [1]. Early and exclusive breastfeeding (within 1 hour of birth), advocated to confer both nutrition and immunity to the newborn, and to facilitate uterine contractions in the mother, was only observed amongst 1 in 3 deliveries and the use of prelacteals was commonplace. Programmes of improved health education with regard to early and essential newborn care may have a positive effect on these practices. Community-based participatory women's group interventions have been found to be particularly effective in implementing behaviour change amongst mothers and potential birth attendants with positive effects on newborn birth outcomes [21-23], and further research is urgently needed as to the effectiveness of this intervention in sub-Saharan Africa.

## Conclusion

Although, in communities such as this, antenatal attendance may be high, there are disparities in the quality of care received and this has an important effect on the standard of care received. Women appear willing and able to travel to health centres offering good high quality services that include the provision of essential services such as VCT and IPTp, and this has important implications for future antenatal health policy and planning. Despite high levels of hospital attendance for delivery, access to essential skilled birth attendants is often difficult especially for less educated, poorer women. Financial and transport limitations are important factors in women's inability to access skilled delivery care and this will need to be addressed if we are to improve delivery service to the most vulnerable of women. Several simple but important delivery and post delivery practices were commonly neglected, including early breast feeding and thermal protection of the newborn, so increasing the risk of morbidity and mortality to mothers and their newborn infants. Community-based strategies with regard to essential newborn care, or participatory interventions through facilitated women's groups, might improve implementation of simple but important neonatal care practices and have positive effects on perinatal behaviour and care seeking.

## Competing interests

The author(s) declare that they have no competing interests.

## Authors' contributions

CT, HG, DM, LM & AE contributed to the study concept and design; MK, CT & MM were responsible for supervision of fieldwork and the acquisition of data; data analysis and interpretation was performed by AE, LM & CT; the manuscript was drafted by CT, AE & LM; the manuscript was revised critically for substantial intellectual content by AE, HG & DM. All authors have reviewed and approved the final version of the paper.

## Pre-publication history

The pre-publication history for this paper can be accessed here:



## References

[B1] World Health Organization (2006). Neonatal and Perinatal Mortality: Country, regional and global estimates.

[B2] Brundtland GH (2002). Perinatal health – a global perspective. J Matern Fetal Neonatal Med.

[B3] Lopez AD (2000). Reducing child mortality. Editorial Bull World Health Organ.

[B4] Black RE (2003). Where and why are 10 million children dying every year?. Lancet.

[B5] Uganda Bureau of Statistics (2001). Uganda Demographic and Health Survey, 2000–2001.

[B6] World Health Organization (2003). Pregnancy, childbirth, postpartum and newborn care: a guide for essential practice.

[B7] World Health Organization, Division of Reproductive Health (1996). Essential Newborn Care, report of a technical working group Trieste, 25–29 April 1994.

[B8] Adam T, Lim SS, Mehta S, Bhutta ZA, Fogstad H, Mathai M, Zupan J, Darmstadt G (2005). Cost effectiveness analysis for maternal and neonatal health in developing countries. Br Med J.

[B9] Tann CJ, Mpairwe H, Morison L, Nassimu K, Hughes P, Omara M, Mabey D, Muwanga M, Grosskurth H, Elliott AM (2006). Lack of effectiveness of syndromic management in targeting vaginal infections in pregnancy in Entebbe, Uganda. Sex Trans Infect.

[B10] Bloom SS, Lippeveld T, Wypij D (1999). Does antenatal care make a difference to safe delivery? A study in urban Uttar Pradesh. India Health Policy and Planning.

[B11] Sai FT, Measham DM (1992). Safe motherhood initiative: getting our priorities straight. Lancet.

[B12] World Health Organization (2003). Antenatal care in developing countries, promises, achievements and missed opportunities: an analysis of trends, levels and differentials, 1990–2001.

[B13] van Eijk AM, Bles HM, Odhiambo F, Ayisi JG, Blokland IE, Rosen DH, Adazu K, Slutsker L, Lindblade KA (2006). Use of antenatal services and delivery care among women in rural western Kenya: a community based survey. Reprod Health.

[B14] van den Broek NR, White SA, Ntonya C, Ngwale M, Cullinan TR, Molyneux ME, Neilson JP (2003). Reproductive health in rural Malawi: a population-based survey. BJOG.

[B15] World Health Organization (1999). Reducing maternal mortality A joint statement by WHO/UNFPA/UNICEF/World Bank.

[B16] World Health Organization (2004). Making pregnancy safer: the critical role of the skilled attendant A joint statement by WHO, ICM and FIGO Dept of Reproductive Health and Research.

[B17] Orinda V, Kakande H, Kabarangira J, Nanda G, Mbonye AK (2005). A sector-wide approach to emergency obstetric care in Uganda. Int J Gynaecol Obstet.

[B18] Kampikaho A, Irwig LM (1991). Incidence and causes of maternal mortality in five Kampala hospital, 1980–1986. East Afr Med J.

[B19] Mbonye AK (2001). Risk factors associated with maternal deaths in health units in Uganda. Afr J Reprod Health.

[B20] Pearson L, Shoo R (2005). Availability and use of emergency obstetric services: Kenya, Rwanda, Southern Sudan and Uganda International. Int J Gynaecol Obstet.

[B21] Wade A, Osrin D, Shrestha BP, Sen A, Morrison J, Tumbahangphe KM, Manandhar DS, de L Costello AM (2006). Behaviour change in perinatal care practices among rural women exposed to a women's group intervention in Nepal [ISRCTN31137309]. Pregnancy Childbirth.

[B22] Howard-Grabman L (1993). The "autodiagnosis": a methodology to facilitate the maternal and neonatal health problem identification and prioritization in women's groups in rural Bolivia, working paper 16A.

[B23] Manandhar DS, Osrin D, Shrestha BP, Mesko N, Morrison J, Tumbahangphe KM, Tamang S, Thapa S, Shrestha D, Thapa B, Shrestha JR, Wade A, Borghi J, Standing H, Manandhar M, Costello AM, Members of the MIRA Makwanpur trial team (2004). Effect of a participatory intervention with women's groups on birth outcomes in Nepal: cluster-randomised controlled trial. Lancet.

